# The oncogenic effects of HES1 on salivary adenoid cystic carcinoma cell growth and metastasis

**DOI:** 10.1186/s12885-018-4350-5

**Published:** 2018-04-17

**Authors:** Xiao-Yu Huang, Rui-Huan Gan, Jian Xie, Lin She, Yong Zhao, Lin-Can Ding, Bo-Hua Su, Da-Li Zheng, You-Guang Lu

**Affiliations:** 1 Department of Preventive Dentistry, Affiliated Stomatological Hospital, Fujian Medical University, 246 Yang Qiao Middle Road, Fuzhou, 350002 China; 2 Key laboratory of stomatology, School of Stomatology, Fujian Medical University, 88 Jiao Tong Road, Fuzhou, 350004 China; 3 Department of Pathology, Affiliated Stomatological Hospital, Fujian Medical University, 246 Yang Qiao Middle Road, Fuzhou, 350002 China

**Keywords:** SACC, RNA-Seq, HES1, Proliferation, Apoptosis, Metastasis

## Abstract

**Background:**

Our previous study demonstrated a close relationship between NOTCH signaling pathway and salivary adenoid cystic carcinoma (SACC). HES1 is a well-known target gene of NOTCH signaling pathway. The purpose of the present study was to further explore the molecular mechanism of HES1 in SACC.

**Methods:**

Comparative transcriptome analyses by RNA-Sequencing (RNA-Seq) were employed to reveal NOTCH1 downstream gene in SACC cells. Immunohistochemical staining was used to detect the expression of HES1 in clinical samples. After HES1-siRNA transfected into SACC LM cells, the cell proliferation and cell apoptosis were tested by suitable methods; animal model was established to detect the change of growth ability of tumor. Transwell and wound healing assays were used to evaluate cell metastasis and invasion.

**Results:**

We found that HES1 was strongly linked to NOTCH signaling pathway in SACC cells. The immunohistochemical results implied the high expression of HES1 in cancerous tissues. The growth of SACC LM cells transfected with HES1-siRNAs was significantly suppressed in vitro and tumorigenicity in vivo by inducing cell apoptosis. After HES1 expression was silenced, the SACC LM cell metastasis and invasion ability was suppressed.

**Conclusions:**

The results of this study demonstrate that HES1 is a specific downstream gene of NOTCH1 and that it contributes to SACC proliferation, apoptosis and metastasis. Our findings serve as evidence indicating that HES1 may be useful as a clinical target in the treatment of SACC.

**Electronic supplementary material:**

The online version of this article (10.1186/s12885-018-4350-5) contains supplementary material, which is available to authorized users.

## Background

SACC is the most common tumor in the minor salivary glands and the second most common tumor in the major salivary glands [1, 2]. Dockerty and Mayo determined that SACC exhibited aggressive features [3]. The disease is characterized by distant metastasis, a high-risk of relapse and a propensity for invading peripheral nerves. Most patients with SACC die within 5 to 20 years of diagnosis [4]. Regional lymph node metastasis was deemed clinically undetectable in affected patients, while hematogenous metastasis to the lungs, bone and liver was frequently reported in patients [5].

NOTCH signaling pathway is a traditional and complex signal pathway, which relates to tissue differentiation and proliferation. There are four NOTCH receptors and five ligands to interactive with each other in mammal. The relationship between disordered NOTCH1 and tumor development has been hotly debated. A volume of reports have demonstrated the role of NOTCH1 acting as oncogene but also tumor suppressor genetically in different cancers. Yuan and colleagues [6] reported that breast cancer patients with NOTCH1 overexpression suffered a low recurrence free survival rate; Arcaroli et al. [7] found that a NOTCH1 gene copy number gain was a worse prognostic in colorectal cancer. On the other hand, several studies reported that high NOTCH1 and NOTCH2 expression with early tumor stages might indicate a tumor-suppressive role of NOTCH signaling in gastric cancer [8]. Intriguingly, hepatocellular carcinoma [9] and medulloblastoma [10] have been observed with both functions even in the same tumor type.

NOTCH is a classical pathway that could activate HES1. Wang et al. [11] found that the expression of NOTCH1 and HES1 was up-regulated consistently in rectal neuroendocrine tumors and pancreatic neuroendocrine tumors. It means that there was a close relationship between NOTCH signaling pathway and HES gene family. HES1 is one of seven members of the HES gene family (HES1–7) [12–14]. HES1 expression is induced by the NICD and encodes a nuclear protein belonging to the hairy and enhancer of related (HESR) family of basic helix-loop-helix (bHLH)-type transcriptional repressors [15–20]. HES1 participates in cellular differentiation, cell apoptosis and cell self-renewal, and the expression level of HES1 is frequently abnormal in cancer cells. Mounting evidences support that HES1 is an oncogene in kinds of tumors. The absence of HES1 has been shown to weaken the tumorigenic capacity of oral squamous cell carcinoma cells [21], as well as colon cancer [22] and pancreatic cancer cells [23].

Our previous study has convincingly verified that NOTCH1 contributed to the cell growth, anti-apoptosis and metastasis of SACC [24]. However, as a complicated signaling pathway, we know little about the influence of this pathway’s upstream or downstream genes in human SACC. Aster’s animal study [25] uncovered harmful side effects by systemic inhibition of NOTCH signaling. So it’s quite important to find the target genes of the pathway and provide a specific means to treat cancer. The objectives of the present study were to illuminate the effects of target gene of NOTCH signaling pathway in human SACC. We analyzed the changes of transcriptome in SACC cells exhibiting NOTCH1 up-regulation by RNA-Seq and verified that HES1 was a specific downstream target of NOTCH1 signaling. HES1 was employed for further study and we compared HES1 expression levels between clinical SACC samples and normal samples using immunohistochemical staining. We also silenced HES1 expression in SACC cell line to determine the effects of HES1 on SACC cell proliferation, migration and invasion and to elucidate the mechanism by which these cells undergo apoptosis.

## Methods

### Cell culture and clinical samples

The SACC cell lines SACC-LM and SACC-83 were obtained from the Peking University Health Science Center. The cells were maintained in RPMI-1640 (Gibco BRL, Grand Island, NY, USA) supplemented with 10% fetal bovine serum (Gibco). The tissue samples were obtained from Fuzhou General Hospital of Nanjing Military Command and Fujian Medical University Union Hospital. Fifty normal salivary tissue samples and 60 SACC samples were used in the study, which were approved by the Institutional Review Board of Fujian Medical University (IRB No. 35000401–11-054). Written informed consent was obtained from each participant.

### RNAi transfection

A negative control (NC) siRNA and two siRNAs against HES1 were synthesized (GenePharma, Shanghai, China). The sequences of these siRNAs are listed in Table 1. The SACC LM cells were transfected with siRNAs using Lipofectamine RNAiMAX (Invitrogen, Carlsbad, California, USA), according to the manufacturer’s instructions.

**Table 1:** The sequences of the siRNAs used in the transfection experiments

Name	Sense	Antisense
siRNA-HES1–425	5’-GGAUGCUCUGAAGAAAGAUTT-3’	5’-AUCUUUCUUCAGAGCAUCCTT-3’
siRNA-HES1–670	5’-CCAACUGCAUGACCCAGAUTT-3’	5’-AUCUGGGUCAUGCAGUUGGTT-3’
NC	5’-UUCUCCGAACGUGUCACGUTT-3’	5’-ACGUGACACGUUCGGAGAATT-3’

### Quantitative real-time PCR analysis

Total RNA was extracted from the SACC LM cells and was reverse transcribed into cDNA. The cDNA was used to detect the expression of the genes of interest by qRT-PCR, which was performed with SYBR Premix Ex Taq (Takara). The primers used in this study are listed in Table 2. The data were analyzed according to the 2^-△△Ct^ method.

**Table 2:** The primers for real-time PCR and semi-quantitative RT-PCR used in this study

Gene	Accession no.	Forward	Reverse
ACTB	NM 001101	CCTGGCACCCAGCACAAT	GGGCCGGACTCGTCATACT
HES1	NM 005524.3	AGGCGGACATTCTGGAAATG	CGGTACTTCCCCAGCACACTT
NOTCH1	NM 017617.3	GGAAGTTGAACGAGCATAGTCC	GCATGATGCCTACATTTCAAGA
CCND1	NM 053056.2	CCCCGCACGATTTCATTGAACA	CATGGAGGGCGGATTGGAAATG
Ki67	NM 002417.4	GCTCCCCACCTCAGAGAGTTTT	CTCTTAAGGGAGGGCTTGCAGA
KRT14	NM 000526.4	GAGCCGCATTCTGAACGAGATG	ACTGCAGCTCAATCTCCAGGTT
IGFBP7	NM 001553.2	GATGCTGGAGAATATGAGTGCC	CCATGACTACTTTTAACCATGCA
PSCA	NM 005672.4	CCAGGTGAGCAACGAGGACT	TAGTCCTGTGAGTCATCCACGC
S100A2	NM 005978.3	ATAAATCCTCACCCTGGGAGCC	CCCTCTTGGCAGGAGTACTTGT
C9ORF3	NM 032823.5	TCTGCGGAAGTGGTGACCC	AGGGTCCAGCTGTATGTCCATG
MMP9	NM 004994.2	GAGGCGCTCATGTACCCTATGT	GGTGTGGTGGTGGTTGGAGG

### RNA isolation, library construction and sequencing

Total RNA was extracted using a mirVana miRNA Isolation Kit (Ambion), according to the manufacturer’s protocol, and RNA integrity was evaluated using an Agilent 2100 Bioanalyzer (Agilent Technologies, Santa Clara, CA, USA). Samples with an RNA Integrity Number (RIN) ≥ 7 were subjected to subsequent analyses. The libraries were constructed using a TruSeq Stranded mRNA LTSample Prep Kit (Illumina, San Diego, California, USA), according to the manufacturer’s instructions. These libraries were then sequenced on an Illumina sequencing platform (HiSeqTM 2500) and 150 bp paired-end reads were generated. Raw data (raw reads) were processed using custom scripts, and ploy-N-containing reads, PCR duplications and low-quality reads were removed to obtain clean reads, which were then mapped to the hg19 genome, which served as a reference, using Tophat (http://ccb.jhu.edu/software/tophat/index.shtml).

### Immunohistochemistry

For the immunohistochemical assays, 5-μm-thick tissue sections were incubated with a primary antibody against HES1 (1:6400, CST, Boston, Massachusetts, USA). All the slides were reviewed independently by two pathologists who were blinded to the other’s readings. The immunohistochemical analysis results were graded with the indicated four-tier scoring system (negative, weakly positive, positive, and strongly positive).

### Western blot assay

Total protein was separated by 8% SDS-PAGE and then transferred onto PVDF membranes (Amersham, Piscataway, NJ, USA), which were immunoblotted with primary antibodies against HES1 (1:1000 dilution, CST) and GAPDH (1:1000 dilution, CST) overnight and incubated with the appropriate secondary antibodies (1:2000 dilution, Abcam, London, UK). The immunoreactive protein bands were visualized using CDP STAR reagent (Roche, Basel, Switzerland).

### Cell viability assay

Cell proliferation was measured by counting viable cells with Cell Counting Kit-8 (CCK-8) (Dojindo, Kumamoto, Japan). The cells were first transfected with siRNAs for 24 h and then plated in a 96-well plate. At the same time during each of the following 5 days, the absorbance of each well was measured at 450 nm with a microplate reader (BioTek, Vermont, USA).

### Colony formation assay

Twenty-four hours after siRNA transfection, the cells were plated in 6-cm plates (600 cells per plate) and cultured for 2 weeks. The colonies were stained with 1% crystal violet.

### Wound healing assay

SACC LM cells were transfected with siRNAs for 24 h after being seeded in a 6-well plate. A 20-μl pipette tip was used to establish a scratch-wound model until the cells amplificated and formed a monolayer covering the bottom of the plate. Then the medium was replaced with 1640 supplemented with 0.1% FBS. The width of scratch-wound was visualized to evaluate the cell invasion ability under a light microscope at the time points of 0 and 72 h and the images were captured.

### Cell invasion and migration assay

Cell invasion was assessed using 24-well Matrigel-coated transwell chambers (8-μm pore size, BD Science, Franklin Lakes, New Jersey, USA). 24 h after siRNA transfection, the cells were serum starved for 24 h and then suspended in 1640 containing 1% FBS. The cells were subsequently plated in the upper transwell chamber at a density of 1.0 × 10^5^ cells/well, and 800 μl of 1640 containing 10% FBS was added to the lower transwell chamber. After incubating for 48 h at 37 °C, the cells in the lower chamber were stained and counted. Cell migration assays were performed with transwell not coated with Matrigel.

### Cell apoptosis and cell cycle assay

Cellular apoptosis was analyzed using an FITC/Annexin V Apoptosis Detection Kit (BD Pharmingen). Cell cycle activity was analyzed using a Cycletest Plus DNA Reagent Kit (BD Pharmingen). The percentage of apoptotic cells and the distributions of the cells in each cell cycle phase were determined using a BD FACS Verse Flow Cytometer.

### Xenograft cancer model

The experimental animal protocol was approved by the Animal Care and Use Committee of Fujian Medical University. Female BALB/c nude mice aged 6~ 8 weeks were purchased from the Center for Animal Experiments of Fujian Medical University. Nude mice were randomly assigned to three groups, each of which comprised 5 mice. The cells (2 × 10^6^) were suspended in 0.2 ml of serum-free 1640 and then injected into the right axillary fossa of each mouse. Tumor size was measured three times a week and was calculated using the formula V = width^2^ × length/2. At the end of the experiment, the tumors were harvested, washed once in PBS, and then weighed.

### Statistical analysis

Statistical analysis of HES1 immunoreactivity was performed using the rank-sum test, and statistical analysis of the PCR and in vitro cell migration/invasion assay results was performed by Student’s t-test. *P* < 0.05 was considered statistically significant, and n.s. was indicated in the figures when *P* > 0.05, * when P < 0.05, **when *P* < 0.01 and *** when *P* < 0.001.

## Results

### HES1 expression is regulated by NOTCH1 in SACC cells

**Fig. 1:**
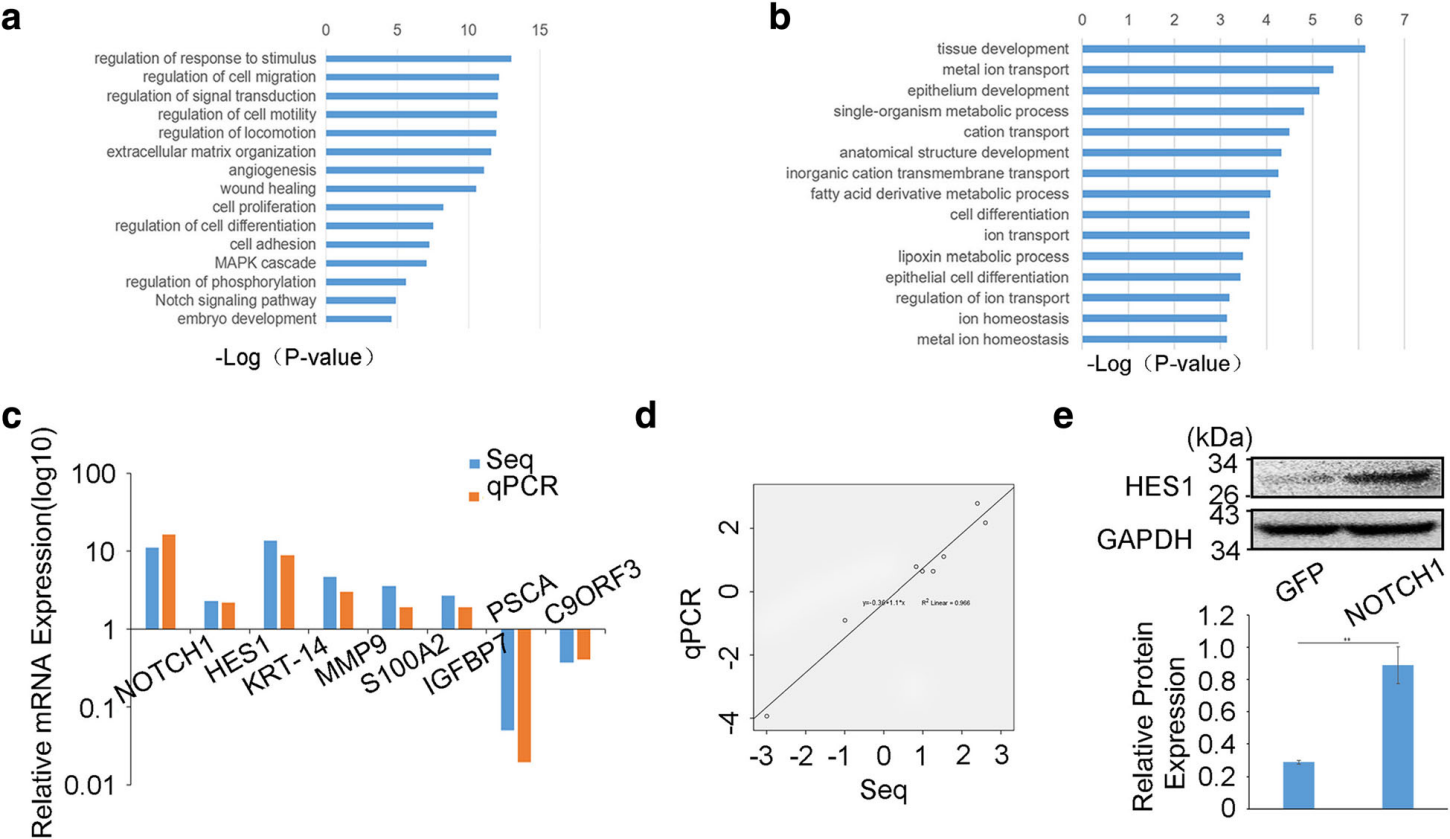
HES1 expression is regulated by NOTCH1 in SACC cells. **a**, **b**, The upregulated genes were associated mainly with cell mobility, cell differentiation and proliferation and signal transduction (**a**), and the downregulated genes were associated with metabolism, transport and differentiation (**b**). **c**, **d**, The expression of NOTCH1, HES1, KRT-14, MMP9, S100A2, IGFBP7, PSCA and C9ORF3 genes, which were selected from the RNA-Seq reports, was measured by qRT-PCR (**c**), and the linear regression analysis was used to compare the expression levels of the abovementioned selected genes between the RNA-Seq results and qRT-PCR results (**d**). **e** Western blot analysis and quantification of HES1 expression in SACC cells after NOTCH1 up-regulation by adenovirus infection

In our previous study, we proved that NOTCH1 acted as an oncogene in SACC and it promoted proliferation and migration of SACC cells [24]. To further explore the effects of downstream gene of NOTCH1 signaling pathway in SACC cells, we performed RNA-Seq to measure the changes of transcriptome after NOTCH1 intracellular domain was overexpressed in SACC-LM cells. Quality control showed a satisfactory quality of RNA with an RIN greater than 8 (Additional file 1: Figure S1 A) and the reads for the duplicate samples were relatively consistent (*R* = 0.989, Additional file 1: Figure S1 B). The RNA-Seq data from this study have been deposited in the NCBI Sequence Read Archive (SRA, https://www.ncbi.nlm.nih.gov/sra) under accession no. SRR5572289. A total of 1323 coding genes displayed differential expression in NOTCH1 overexpressed cells, including 806 upregulated coding genes and 517 downregulated coding genes (Additional file 1: Figure S1C, D). Gene Ontology (GO) analysis provided a measure of critical function, which showed that the upregulated genes were associated mainly with cell mobility, cell differentiation, proliferation and signal transduction, while the downregulated genes were associated with metabolism, transport and differentiation (Fig. 1a, b).

Based on the RNA-Seq results, we selected seven deregulated genes randomly along with NOTCH1 to validate the expression by qRT-PCR. The expression level of HES1, KRT-14, MMP9, S100A2, IGFBP7, PSCA and C9ORF3 by qPCR was consistent with the results of RNA-Seq (Fig. 1c, *P* < 0.001), which proved that HES1 was one specific downstream gene of the NOTCH1 signaling pathway. The linear regression analysis towards to the relationship between RNA-Seq and qPCR displayed a reliable result of RNA-seq (Fig. 1d, R^2^ = 0.966). Furthermore, we tested the expression of HES1 by western blot analysis (Fig. 1e), and the SACC cells transfected with NOTCH1 overexpressed plasmid displayed a higher expression of HES1 compared with control group. Additionally, the expression of HES1 has been verified decreased while NOTCH1 was restrained in SACC cells in our previous study [24]. In conclusion, we confirmed the reliability of our RNA-Seq results and focused on the potential downstream gene of NOTCH1 signaling pathway, HES1, for a series of further study.

### HES1 is up-regulated in metastatic and recurrent adenoid cystic carcinomas

**Fig. 2:**
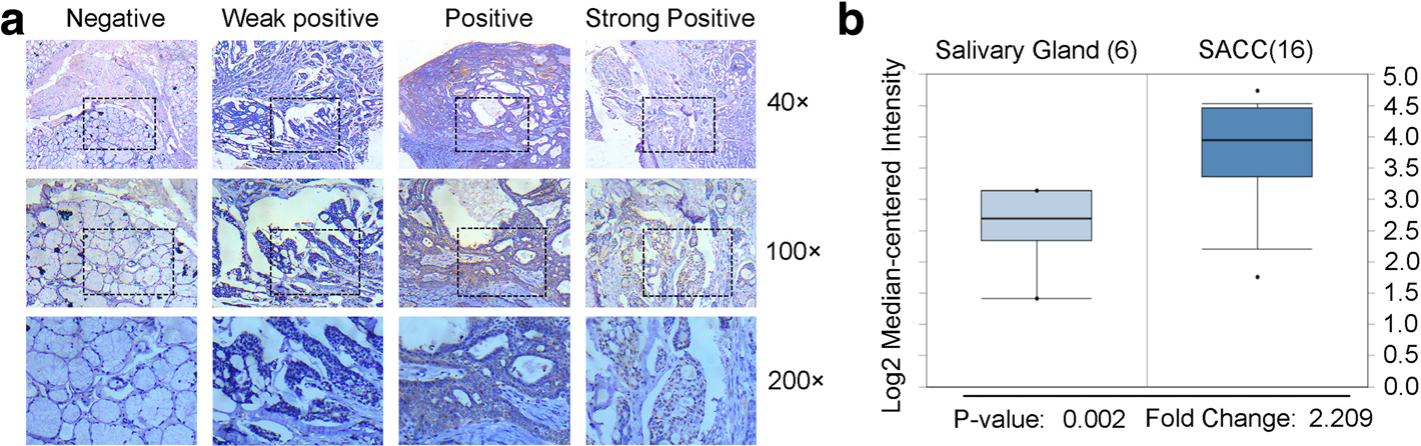
HES1 is up-regulated in metastatic and recurrent adenoid cystic carcinomas. **a** Representative images of HES1 expression, as determined by immunohistochemistry and the following four-tier scales: negative, weakly positive, positive and strongly positive. **b** The mRNA expression of HES1 was upregulated in the SACC samples compared with salivary glands, according to the Frierson dataset from the Oncomine database, the fold-change was 2.209 (*P* = 0.002)

Basing on the results of RNA-Seq and our previous data, we explored the expression of HES1 by immunohistochemistry to validate its role in SACC. A total of 50 normal tissue samples and 60 SACC tissue samples were included in the study. We classified HES1 expression on a four-tier scale, according to the staining results. As shown in Fig. 2 and Table 3, HES1 expression was absent or very low in normal salivary gland tissues; however, HES1 expression was high in adenoid cystic carcinoma tissues (*P* < 0.001). The clinical stages of SACC patients were divided into early and late stages by TNM classification. Further analysis of clinical information revealed that HES1 expression was higher in late stages (tumor size>4 cm, or with lymph node or distant metastasis) than that in early stages (tumor size ≤4 cm, or without lymph node or distant metastasis) (P < 0.001, Table 4), and is especially higher in that with lymph node or distant metastasis (*P* < 0.05) than without. Additionally, we searched the expression level of HES1 in the publicly available Oncomine database and found that HES1 was upregulated in SACC as showed by Frierson HF, who assessed the expression of 8603 genes in 22 samples by microarray (Fig. 2b) (http://www.oncomine.org) [26]. These results indicated that HES1 might participate in the initiation and progress of SACC.

**Table 3:** HES1 expression in normal salivary and SACC tissues

Samples	Cases	Negative	Weakly positive	Positive	Strongly positive	*P*-value
Normal Salivary	50	43	7	0	0	
SACC	60	0	48	11	1	< 0.001

Rank-sum test Z = −5.699

**Table 4:** The expression of HES1 in clinical and pathological characteristics of SACC

Characteristics	Total	Low HES1^a^	High HES1^a^	*P* value
Gender				
Female	23	19	4	0.693
Male	37	29	8	
Age				
≤55	29	25	4	0.249
> 55	31	23	8	
Stage				
Early	19	32	1	0.001***
Late	14	16	11	
Invasion				
No	28	25	3	0.095
Yes	32	23	9	
Metastasis (Lymph node and distant)				
No	52	44	8	0.024**
Yes	8	4	4	

^a^Because of limited SACC clinical samples, the expression of HES1 was divided into two levels, in which low expression included the negative and weakly positive as shown in Fig. 3 and Table 3, and high expression included positive and strongly positive

** *P* < 0.05. ***P < 0.01

### HES1 regulates cellular apoptosis in vitro

**Fig. 3:**
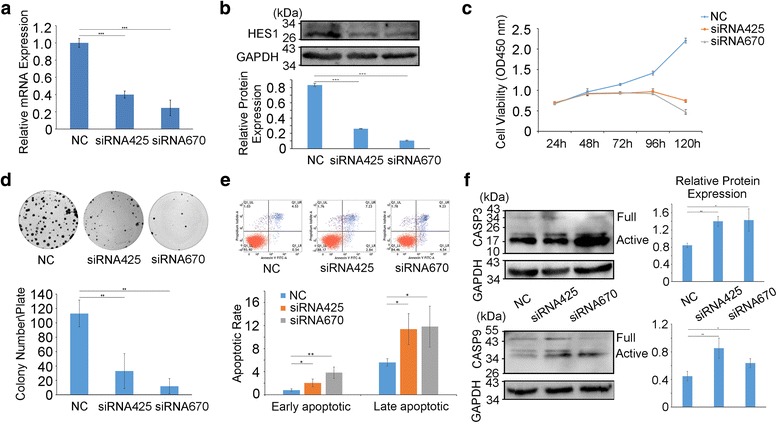
HES1 promotes cell proliferation and regulates cellular apoptosis in vitro. **a**, **b** Forty-eight hours after siRNA transfection, HES1 expression in SACC cells was measured by real-time PCR (**a**) and western blotting (**b**). **c**, **d** After siRNA transfection, SACC cell proliferation was detected by CCK-8 (C, *P* < 0.001 on days 3, 4 and 5) and colony formation assay (**d**). **e** The percentages of early (Annexin V-positive and PI-negative) and late-apoptosis cells (Annexin V- and PI-positive) were analyzed by flow cytometry. F, The expression of the apoptosis-related genes CASP3 and CASP9 was measured by western blotting in HES1-knockdown cells

To investigate the effects of HES1 on cellular apoptosis, we knocked down HES1 via siRNA in SACC cells. The qRT-PCR (Fig. 3a) and western blotting (Fig. 3b) results showed that the siRNAs targeting HES1 (siRNA-425 and siRNA-670) efficiently reduced HES1 expression in SACC cells compared with negative control (NC) cells. After the cells were transfected with these siRNAs, their growth was significantly inhibited, as demonstrated by CCK8 assay (Fig. 3c, *P* < 0.001 on day 3, 4 and 5). Similar results were noted in the colony formation assays (Fig. 3d, *P* < 0.01, *n* = 3). To explore the effects of HES1 on cancer further, we knocked down HES1 via siRNA transfection for 48 h and then quantified the numbers of apoptotic cells via Annexin V and PI staining and flow cytometric analysis. After 48 h of transfection, the percentages of cells undergoing (Fig. 3e) early (Annexin V-positive and PI-negative) and late apoptosis (Annexin V-positive and PI-positive) were higher among HES1-silenced cells than among control cells. We performed western blotting to detect CASP3 and CASP9 expression in HES1-knockdown cells and full-length and cleaved bands were observed. Through quantification of the active bands, we concluded that the cleaved CASP3 and CASP9 protein levels (Fig. 3f) were elevated in the indicated group of cells compared with NC cells. At the same time, we also applied the PI staining flow cytometry cycle tests to explore whether HES1 knockdown affected the cell cycle phases. The results didn’t show consistent trend and there was not significant difference between NC and HES1 siRNAs (Additional file 1: Figure S2). Collectively, these results confirmed that knocking down HES1 promoted cell apoptosis in vitro, which indicated that HES1 played an oncogenic role in SACC.

### HES1 knockdown inhibits tumorigenicity in vivo

**Fig. 4:**
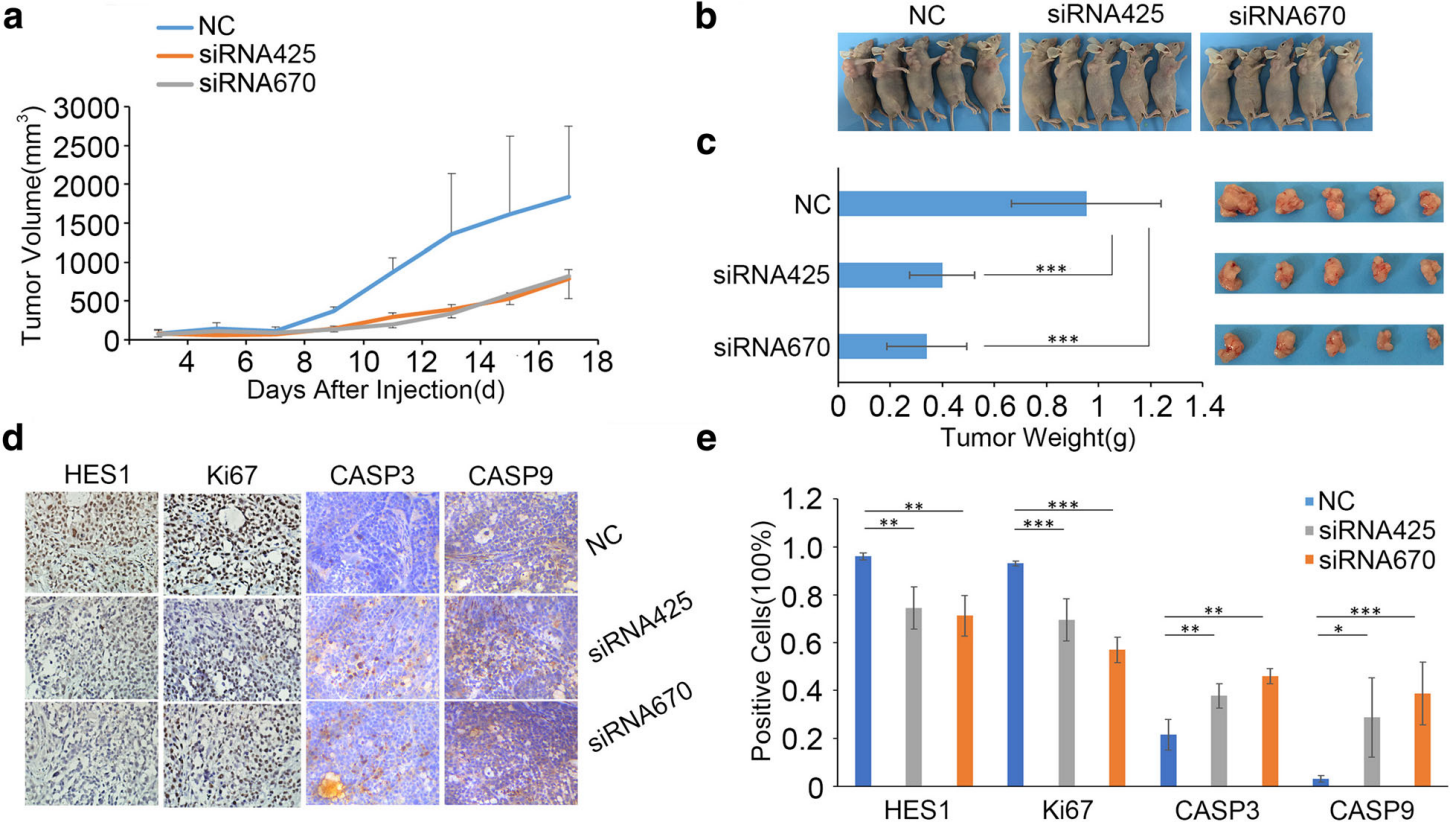
HES1 knockdown inhibits tumorigenicity in vivo. **a** After being transfected with siRNA, the SACC cells were subcutaneously injected into the flanks of nude mice (*n* = 5), and the sizes of the resultant tumors were measured three times per week using a digital caliper. **b** The mice were placed and photographed; **c** The tumors were excised, photographed and weighed. D-E, The expression of HES1, Ki67, CASP3 and CASP9 was detected in the xenograft tumors by immunohistochemistry (**d**) (DAB, 400×) and the positive cells were counted and compared among the groups (**e**)

To explore the effects of HES1 on tumorigenicity in vivo, we transfected SACC LM cells with HES1-siRNAs to silence endogenous HES1 and then subcutaneously inoculated the cells into the flanks of athymic mice. HES1 knockdown inhibited tumor growth, as determined by our results pertaining to xenograft tumor size (Fig. 4a, b) and tumor wet weight (Fig. 4c), in xenografts comprising cells transfected with siRNA425 and siRNA670 compared with xenografts comprising control cells. Moreover, we detected and analysethe expression of HES1, Ki67, CASP3 and CASP9 in the xenograft tumors tissues using immunohistochemistry (Fig. 4d, e). The immunohistochemistry expression of HES1 in the siRNA425 and siRNA670 transfected xenograft tumors groups was lower than in the control group, which supported the effective transfection with siRNA after inoculation for 18 days. The statistical proliferative index of tumor tissues stained for Ki67 came to the conclusion that HES1 knockdown also affected cell proliferation in vivo. We considered that HES1 downregulation in SACC cells led to decreases in cell proliferation and increases in cell apoptosis and apoptosis-related protein expression among the transfected groups compared with the control group, indicating that reductions in HES1 expression in SACC cells could induce cellular apoptosis to inhibit cell growth.

### HES1 increases cell migration and invasion in vitro

**Fig. 5:**
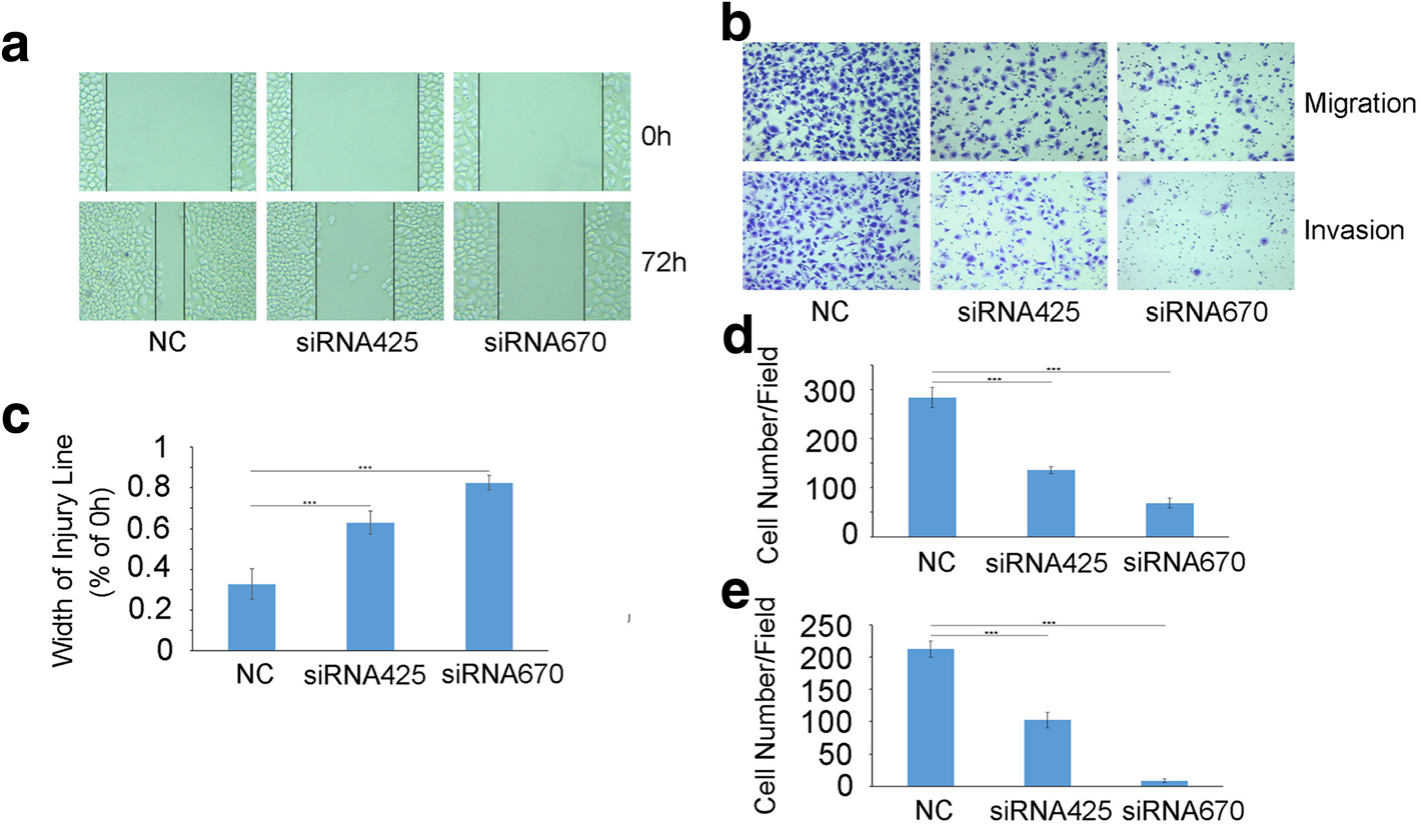
HES1 knockdown inhibits SACC cell migration and invasion ability. **a**, **b** A photomicrograph of the scratch wounds made in the siRNA-transfected SACC cell layer showing that cellular motility was inhibited in HES1-silenced cells compared with control cells (**a**) and the width of injury line after transfection for 72 h compared with 0 h was quantificated (**b**). **c** Representative images of transwell chambers coated without (upper panel) or with (lower panel) Matrigel after siRNA transfection. **d** The number of cells that migrated through the uncoated filters (i.e., no Matrigel), which represents the migratory ability of the indicated SACC cells. **e** The number of cells that were able to pass through the filters precoated with Matrigel, which represents the invasive ability of the indicated SACC cells. The cell counts are presented as the mean number of cells per field from at least five randomly selected low-powered fields (200×) from three independent experiments (error bars, means ± SD)

We subsequently assessed the effects of knocking down HES1 on SACC migration and invasion. Transfecting HES1-specific siRNAs into SACC cells significantly inhibited SACC cell motility and invasiveness, as demonstrated by wound healing (Fig. 5a, b) and transwell assays (Fig. 5c, d and e *P*< 0.05, *n* = 3). These results supported the idea that HES1 acted as an oncogene in SACC and contributed to adenoid cystic carcinoma cell migration and invasion.

## Discussion

There are emerging studies regarding SACC molecular biology. The genetic markers differentially expressed between cancerous cells and normal cells are molecules related to cell proliferation [27], growth factor receptors and ligands [28, 29], cell cycle oncogenes [30, 31], cell adhesion proteins [32], and transcription factors [33, 34]. However, HES1 has never been reported to play a role in SACC. HES1 encodes nuclear proteins to activate transcription repression in two ways [13, 35]. One is to form a non-DNA binding complex by joining with other bHLH factors via the bHLH domain, and another is to cooperate with co-repressor transducin-like enhancer of split (TLE) to prevent itself from binding to the N box through its WRPW motif and forming complexes [36, 37]. HES1 induction may depend on several disease-specific and cell-dependent signaling pathways, such as the NOTCH pathway [38], the hedgehog pathway [39, 40], the c-Jun N-terminal kinase (JNK) signaling pathway [41, 42] and the MAP kinase ERK pathway [43]. These pathways appear to be involved in cross-talk with one another at the molecular level. NOTCH is a canonical pathway in the abovementioned pathways, and HES1 plays a prominent role in the NOTCH-HES1 axis. HES1 expression was activated upon the induction of the transcriptional complex CSL-NICD in the nucleus [44]. The NOTCH signaling pathway has been reported to link various microenvironmental factors to the occurrence and development of malignant tumors. Following our previous study regarding the role of the NOTCH1 signaling pathway in SACC, we devoted our attention to systemically studying NOTCH1 [24]. In this study, we revalidate HES1 as the definite downstream gene of NOTCH1 in SACC cells via RNA-Seq analysis. Immunochemistry showed that HES1 expression levels in SACC cancerous tissues were much higher than in those in para-cancerous tissues, too. All of these give us clues that HES1 is a valuable target gene of NOTCH1 signaling pathway and captivate us to carry out more research in SACC.

Cell proliferation is a recognized key contributor to the rapid development of cancer. And stem cells are the source of cancer cells endless proliferation. Many studies indicate that HES1 has the potential to induce cancer stem cells with self-transforming ability and to trigger apoptosis resistance and oncogenesis progression. In addition, the NOTCH-HES1 pathway was verified to affect stem cell maintenance in breast cancer [45]. Our study found that the cells growth ability of SACC cells was affected by HES1. As for illuminating the relationship between HES1 and the stem cells of SACC, we will need an in-depth study in the future. It has also been reached to a consensus that HES1 is associated with apoptosis [22]. Cancer cell differentiation and apoptosis can be stimulated via NOTCH and hedgehog pathway inhibition. It is suspected that differentiation is suppressed by HES1-mediated histone deacetylase (HDAC) inhibition. HDACs have been shown to induce differentiation or apoptosis in tumors and may thus be useful as anti-tumor therapeutic agents [46]. HES1 downregulation induces growth arrest and apoptosis in acute myeloid leukemia (AML) cells; thus, HES1 may be a novel target for the treatment of AML [47]. The execution-phase of cell apoptosis can be sequentially activated by CASP. CASP3 and CASP9 are common members of the CASP family and interact with each otherCASP9 can process and activate CASP3 [48]. To explore the relationship between HES1 downregulation and apoptosis inducement in SACC cells, flow cytometry was involved in our research. It provided new insight that HES1 shared a close association with apoptosis by attracting our attention on apoptosis-related genes, such as CASP3 and CASP9, which might induce apoptosis in SACC cells.

The fate of invasion and metastasis is associated with epithelial-to-mesenchymal transition (EMT), as previous studies have shown that EMT is triggered in metastatic prostate cancer PC3 cells [49]. Otherwise, Fei Gao [22] demonstrated that EMT was enhanced by HES1, which facilitates colon cancer cell aggressiveness and inhibited by HES1 silencing. Weng MT found that HES1 controls invasiveness via the STAT3-MMP14 pathway in colorectal cancer (CRC) cells [50]. Our research also implied that HES1 promoted SACC cells migration and invasion ability, and the clinical samples analysis indicated that the high expression of HES1 in SACC might relate to more metastasis.

It is noteworthy that, the cell or tissue samples and data mining we conducted in the study were insufficiency, so larger sample size would be attempted in the future. In the meanwhile, further studies are needed to clarify specific molecule mechanism of the proliferation and metastasis of HES1 in SACC.

## Conclusions

We have determined that HES1 played a vital role in maintaining metastasis, proliferation and apoptosis in SACC cells; thus, targeting HES1 may represent a promising means by which SACC can be treated. In addition to playing a role in the NOTCH signaling pathway, treatments targeting HES1 may cause fewer side effects than those targeting whole NOTCH signaling pathway totally. Therefore, clinicians and researchers should devote significant attention to developing clinical therapies designed to target HES1.

## Additional file

Additional file 1: **Figure S1**. RNA-sequencing for Notch1 over-expressing cells. A, The electrophoresis results of RNA extracted from the SACC cell samples implied a satisfactory RNA quality.1 N1 was the cells sample with NOTCH1 overexpressed vector while 1PS was the cells sample with negative vector, 3 N1 and 3PS were the duplicate samples of 1 N1 and 1PS. B, The reads of RNA-seq showed highly consistent in duplicate samples. C, Volcano plot for RNA-sequencing. Differentially expressed genes in each conditions were identified (in red or green), which is statistically significant. D, Heatmap for differentially expressed genes. **Figure S2**. The effect of HES1 knockdown on cell cycle. A, Representative images of Flow Cytometry analysis of cell cycle after PI staining. B, The percentage of cells in each phase of the cell cycle. Data from 2 independent experiments. (PDF 213 kb)

## Abbreviations

AML: Acute myeloid leukemia
bHLH: Basic helix-loop-helix
CASP3: Caspase 3
CASP9: Caspase 9
CCK8: Cell Counting Kit-8
CRC: Colorectal cancer
EMT: Epithelial-to-mesenchymal transition
GO: Gene Ontology
HDAC: HES1-mediated histone deacetylase
HES: Hairy and enhancer of split
JNK: c-Jun N-terminal kinase
NC: Negative control
NICD: NOTCH intraCellular domain
qRT-PCR: Quantitative real-time PCR analysis
RIN: RNA Integrity Number
RNA-Seq: RNA-Sequencing
SACC: Salivary adenoid cystic carcinoma
SRA: Sequence Read Archive
TLE: Transducin-like enhancer of split
