# Supplementary material for: The oncogenic effects of HES1 on salivary adenoid cystic carcinoma cell growth and metastasis
Source: BMC Cancer. 2018 Apr 17;18:436. doi: 10.1186/s12885-018-4350-5 (PMC5904989; doi:10.1186/s12885-018-4350-5)
Supplement: Supplementary file 1 — Figure S1. RNA-sequencing for Notch1 over-expressing cells. A, The electrophoresis results of RNA extracted from the SACC cell samples implied a satisfactory RNA quality.1 N1 was the cells sample with NOTCH1 overexpressed vector while 1PS was the cells sample with negative vector, 3 N1 and 3PS were the duplicate samples of 1 N1 and 1PS. B, The reads of RNA-seq showed highly consistent in duplicate samples. C, Volcano plot for RNA-sequencing. Differentially expressed genes in each conditions were identified (in red or green), which is statistically significant. D, Heatmap for differentially expressed genes. Figure S2. The effect of HES1 knockdown on cell cycle. A, Representative images of Flow Cytometry analysis of cell cycle after PI staining. B, The percentage of cells in each phase of the cell cycle. Data from 2 independent experiments. (PDF 213 kb) [file 12885_2018_4350_MOESM1_ESM.pdf]

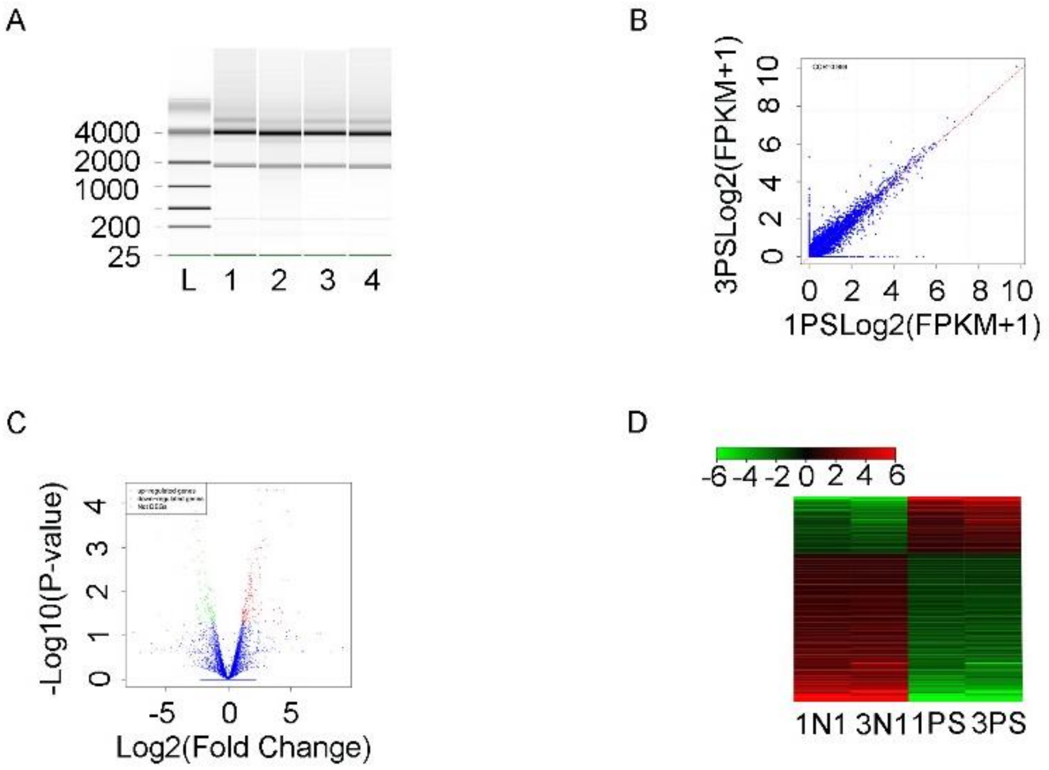

Figure.S1. RNA-sequencing for Notch1 over-expressing cells. A, The electrophoresis results of RNA extracted from the SACC cell samples implied a satisfactory RNA quality.1N1 was the cells sample with NOTCH1 overexpressed vector while 1PS was the cells sample with negative vector, 3N1 and 3PS were the duplicate samples of 1N1 and 1PS. B, The reads of RNA-seq showed highly consistent in duplicate samples. C, Volcano plot for RNA-sequencing. Differentially expressed genes in each conditions were identified (in red or green), which is statistically significant. D, Heatmap for differentially expressed genes.

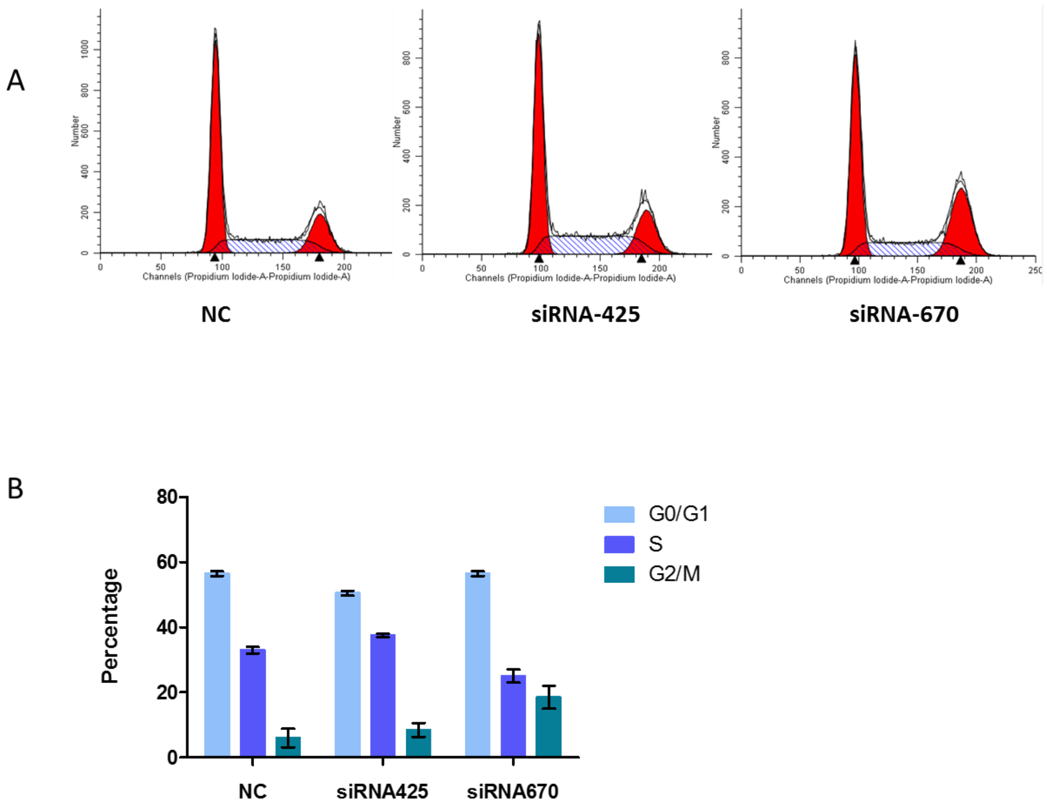

Figure.S2. The effect of HES1 knockdown on cell cycle. A, Representative images of Flow Cytometry analysis of cell cycle after PI staining. B, The percentage of cells in each phase of the cell cycle. Data from 2 independent experiments.
